# The Impact of Colchicine on COVID-19 patients: A Clinical Trial Study

**DOI:** 10.31138/mjr.33.2.232

**Published:** 2022-06-30

**Authors:** Farhad Salehzadeh, Farhad Pourfarzi, Sobhan Ataei

**Affiliations:** 1 Pediatric Department, Bouali Children`s Hospital, Ardabil University of Medical Sciences (ARUMS), Ardabil, Iran,; 2 Department of Community Medicine, Ardabil University of Medical Sciences (ARUMS), Ardabil, Iran,; 3 Pediatric Department, Bouali Children’s Hospital, Ardabil University of Medical Sciences (ARUMS), Ardabil, Iran

**Keywords:** COVID-19, colchicine

## Abstract

**Background:**

Severe acute respiratory syndrome due to COVID-19 infection has evolved into a global pandemic. This study has been designed to evaluate colchicine as an anti-inflammatory agent among COVID-19 patients regarding the disease course, duration of hospitalisation, and its morbidity and mortality rate.

**Methods:**

This prospective randomised and double-blind clinical trial study included 100 COVID-19 hospitalized patients with moderate symptoms from May 21 to June 20, 2020. They were randomised in a 1:1 allocation to placebo and colchicine groups plus recommended standard guideline and protocol of health system. Colchicine 1 mg has been taken daily for 6 days. All data including associated symptoms, co-existed disease and duration of hospitalisation evaluated initially, and then 2 weeks after discharge; moreover, their mortality and morbidity, re-admission, and deteriorations of symptoms were assessed during this period.

**Results:**

59% were female with median age 56 years old. There was no significant difference between them in terms of age and sex. Two groups did not show significant difference about underlying diseases and various clinical and para clinical findings evaluation. However, there were significant difference in colchicine group regarding for shorter duration of fever (P<0.05) and hospitalisation (P<0.05). Although in colchicine group dyspnoea improved more rapidly than the placebo group, it was not meaningful.

**Conclusion:**

Colchicine can be effective in amelioration of systemic symptoms and duration of hospitalisation probably by inhibition of inflammatory biomarkers in COVID-19 patients.

## BACKGROUND

In late 2019, several cases of an acute respiratory illness (now known as the new coronavirus or COVID-19) were reported in Wuhan, China.^1–3^ The coronavirus has spread rapidly to all over the world. As of Dec. 1, 2020, a total of over 61.8 million people infected by the virus and caused 1.4 million deaths all over the world.^4^ There are various reports on the pathophysiology of the disease. Many studies have suggested that an over-reaction of the immune system by virus can cause the complicated features of disease.^5^ Cytokine storm syndrome is the severe immune reaction that may cause a severe tissue response in these patients.^6^ By innate immune system stimulation, NLRP3, an inflammasome compartment activates IL-18, IL-1β, and IL-6.^7^ Excessive synthesis of IL-6 against infection leads to an acute systemic inflammatory reaction known as cytokine storm.^8^

IL-6, which plays the main role in cytokine storm, is produced by activated leukocytes causing excretion of several other cytokines subsequently. On the other hand, the production of these cytokines is mainly triggered in order to develop an inflammation to suppress the infection.^9^ Considering the role of inflammation in both exacerbation and suppression of the disease, it can be hypothesised that altered mechanisms of innate immunity pathway as a main role in IL-6 production may result in different clinical features of the disease. Acute lung injury and even acute respiratory distress syndrome (ARDS) are common outcomes of cytokine storm in lung alveolus.^10^

Colchicine is a drug that is widely used to treat and prevent acute crystal arthropathies, Familial Mediterranean Fever (FMF), and systemic vasculitis such as Behçet’s disease. In addition, it has been shown that colchicine is an important drug in inflammatory diseases due to its widespread anti-inflammatory effects, particularly by stabilising of polymorphonuclear cells (PMN).^11^

Colchicine affects NLRP3 and prevents the activation of IL-18, IL-1β and IL-6,^7^ and appears to play a significant role in reducing and controlling of the cytokine storm. According to its anti-inflammatory effects, it seems that Colchicine may have a significant effect on improving the symptoms, course and mortality rate caused by the new coronavirus disease. Therefore, this double-blind clinical trial study has been conducted to evaluate the effect of this drug on the symptoms, duration of hospitalisation, and mortality rate among COVID-19 patients.

## METHODS

### Patients and data

This study took place from May 21, 2020 until June 20, 2020 at Imam Reza Hospital in Ardabil city, in northwest Iran. A randomised, double-blind clinical trial study among 100 adult patients with COVID-19 was conducted. Including criteria were defined as pulmonary involvement in CT-Scan which compatible with COVID-19 and Positive PCR of COVID-19. Excluding criteria were defined as follows: hypersensitivity to any medications of therapy, renal failure, heart failure, pregnancy, participating in another clinical study, and refusal to participate in the study before or during the follow-up period.

### Treatment regimens

This is a prospective, randomised clinical trial, controlled group study. Patients were randomised in 1:1 allocation in two equal groups (group-A and group-B) which contains 50 patients. Patients of group-A were treated by colchicine (1 mg daily for six days / Mofid Pharma.co) plus health care system guideline (first protocol of health department was issued in March 2020), while group-B patients were taken placebo plus same protocol. This protocol included supportive therapy such as IV hydration, oxygen, Azithromycin, Hydroxychloroquine, and Naproxen for all patients during first week.

### Study designs, and assessment

Clinical assessments were evaluated after admission and as a daily re-examination program then 2 weeks after discharge. This study adheres to CONSORT guidelines and include a completed CONSORT checklist. All patients of the study filled confirmed consent form.

### Statistical Analysis

In this study, SPSS statistical analysis software version 25 was used to analyse the data. Because of the number of cases (more than 30), we assume that it has normal distribution. The data were first expressed using the frequency amount (number, percentage, mean), and then using independent T-test and chi-square test; the relationship between them was later examined, and the results were presented in tables. Amounts less than 0.05 were considered meaningful findings. Current Controlled prospective Trials registration ID approved by ICMJE and WHO ICTRP registry is IRCT20200418047126N1, and the date of registration is 2020-05-14. The domestic ethical code was IR.ARUMS.REC.1399.050.

## RESULTS

Among 100 patients, 59% were female with median age, 56 years old. Their basic characteristics are summarised in **Table 1**.

**Table 1.** Basic characteristics of patients in two groups.

**Characteristic**	**No. (%)**		P Value*
	**Colchicine (n=50)**	**Placebo (n=50)**	
Male	19(38)	22(44)	0.542
Female	31(62)	28(56)	
Age (mean/year)	56.56 ± 17.16	55.56 ± 16.38	0.766
Time from suffering to Enrolment (mean/day)	6.28 ± 2.51	8.12 ± 2.66	**0.001**
**Co-existing disease:**			
Diabetes Mellitus	5(10)	6(12)	0.749
Ischemic Heart Disease	6(12)	9(18)	0.401
Hypertension	3(6)	8(16)	0.110
Cancer/Neoplastic Disorder	1(2)	1(2)	1.000
COPD	0(0)	4(8)	**0.041**
Renal failure	4(8)	1(2)	0.169
Hypothyroidism	1(2)	1(2(	1.000
Symptoms:			
Fever	34(68 )	39(78)	0.260
Myalgia	18(36)	22(44)	0.414
Cough	30(60)	33(66)	0.534
Dyspnoea	21(42)	18(36)	0.539
Vomiting	6(12)	2(4)	0.140
Nausea	8(16)	9(18)	0.790
Sweating	4(8)	9(18)	0.137
Headache	7(14)	9(18)	0.585
Laboratory:			
White blood cell count, mean,/μL	6544 ± 3132.98	6486 ± 2938.83	0.924
Neutrophil count/μL	4916.14 ± 2589.81	4894.66 ± 2846.84	0.969
Lymphocyte count/μL	1136.40 ± 463.36	1255.62 ± 664.56	0.304
Eosinophil count/μL	189.78 ± 131.53	269.82 ± 305.49	0.088
Monocyte count/μL	100.12 ±73.29	90.80 ± 61.61	0.532
Haemoglobin, mean, g/dL	12.81 ± 2.48	12.91 ± 2.53	0.837
Platelet, mean, ×10^3^/μL	192 ± 63	200 ± 82	0.558
Creatinine, mean, gr/dL	1.23 ± 0.68	1.06 ± 0.42	0.262
ESR, mean, mm/hr	37.73 ± 23.69	43.13 ± 19.87	0.256
C-reactive protein (CRP), (1+,2+&3+), mg/L	1.80	1.63	0.886
AST, mean,/L	35.25 ± 23.37	114.22 ± 510.32	0.287
ALT, mean,/L	40.48 ± 39.58	115.28 ± 499.46	0.304
Alk.P, mean,/L	179.63 ± 137.48	185.51 ± 129.45	0.837
PT, mean,/s	14.38 ± 2.04	15.33 ± 3.84	0.319
PTT, mean,/s	35.32 ± 6.02	33.30 ± 4.08	0.215
INR, mean	1.06 ± 0.07	1.21 ± 0.59	0.258

* The significance level is less than 0.05

**Table 2.** Post-discharge findings of two groups.

**Characteristic**	**No. (%)**		P Value *
	**Colchicine (n=50)**	**Placebo (n=50)**	
Duration of hospitalised, /day	6.28 ± 2.51 8.12 ± 2.66		0.001
Diabetes Mellitus	5(10)	6(12)	0.749
Ischemic Heart Disease	6(12)	9(18)	0.401
Hypertension	3(6)	8(16)	0.110
Cancer/Neoplastic Disorder	1(2)	1(2)	1.000
COPD	0(0)	4(8)	0.041
Renal failure	4(8)	1(2)	0.169
Fever	1(2)	11(22)	0.02
Myalgia	2(4)	4(8)	0.400
Cough	2(4)	2(4)	1.000
Dyspnoea	3(6)	6(12)	0.295
Vomiting	0(0)	2(4)	0.153
Nausea	4(8)	3(6)	0.695
Sweating	0(0)	0(0)	-
Headache	1(2)	3(6)	0.307

* The significance level is less than 0.05

There is no significant difference between the two groups in terms of age and sex. Two groups did not show meaningful difference regarding underlying diseases, except for COPD. In view of various clinical and para-clinical findings during admission and post-discharge evaluation there was no valuable difference. However, there was significant difference in colchicine group regarding for shorter persistence of fever (P<0.05) and duration of hospitalisation (P<0.05). Although in colchicine group, dyspnoea improved more rapidly than the placebo group, it was not meaningful.

## DISCUSSION

There is no specific treatment for COVID-19, although some antiviral agents and biologic drugs have been used with different success. In respiratory failure, non-invasive and or invasive mechanical ventilation may be required.^12^ Convalescent plasma and/or immunoglobulin have been used as alternative resort to improve the survival rate of patients with COVID-19 whose condition continued to deteriorate, despite treatment with methylprednisolone pulse therapy.^13^

5% of infections with COVID-19 complicated by acute respiratory distress syndrome (ARDS), which required mechanical ventilation. Concerning ARDS treatment, it seems plausible to speculate that the anti-IL6 plays a protective role if given at the time of overly elevated immune response to the virus, thus preventing “anaphylactic toxicity”. Such extreme cytokine reaction is accompanied by infiltration of inflammatory monocytes/macrophages into the lung and elevated production of the pro-inflammatory cytokines.^14^

Colchicine has been shown to limit IL-1b production as a response to various NLRP3 inflammasome inducers in a dose-dependent form. For example, in the setting of acute coronary syndrome, colchicine was effective in suppressing interleukin IL-1b, IL-18 and IL-6, which was attributed to inflammasome inhibition.^15,16^

Initially, hydroxychloroquine has been found to be effective against COVID-19,^17^ and on the basis of this data, it had been included in various early guidelines as an anti-inflammatory agent. In this study in terms of symptoms, and during the follow-up, fever was decreased significantly in colchicine group (P=0.02). Moreover, the hospitalised period was significantly shorter in this group (P=0.001). Although, in colchicine group dyspnoea subsided more rapidly than the placebo group, it was not meaningful. None of the patients died or were readmitted. In Spyridon et al. study about the colchicine effect on cardiac and inflammatory biomarkers in COVID-19 admitted patients, mean (SD) event-free survival time was 18.6 (0.83) days in the control group vs. 20.7 (0.31) in the colchicine group (log rank P = 0.03).^18^

In Mansouri et al. study,^19^ they described the case of a 42-year-old healthy patient with COVID-19 who despite improvement in his respiratory symptoms developed a mild to moderate cytokine release syndrome (CRS) and an associated monoarticular gout flare. Since the patient refused admission to the hospital and had stable vital signs, they treated him with a safe anti-inflammatory and non-immunosuppressive therapy. To hit two birds with one stone, they considered colchicine, as it has systemic anti-inflammatory effects and is also effective in gout flare. Unexpectedly, 48 hours after treatment, not only did his ongoing fever and toe pain disappear, he also had significant improvements in his general state of health and all his inflammatory markers including fibrinogen, ferritin, D-dimer, and IL-6 levels normalised.

The inflammatory basis of the COVID-19 that includes cytokine storm syndrome because of excessive synthesis of IL-6 and also due to the anti-inflammatory effects of colchicine on the innate immune system by stabilizing of PMNs^15,16^; it seems colchicine could be effective in improving systemic COVID-19 symptoms such as fever, which is undoubtedly induced by inflammatory biomarkers, eg, IL-6 and TNF (tumour-necrosis factor). Inhibition of these biomarkers by colchicine may prevent acute respiratory syndrome and lead to suppression of cytokine storm progressiveness, which is the most dangerous event in COVID-19.^6,8^ However, it is necessary to confirm these results with further studies.

## LIMITATIONS

This study was performed only on the clinical aspects of the patients and the changes in biomarkers were not considered, moreover it was performed only in non-ICU and moderately symptomatic patients to evaluate its preventive ability in disease course.

## CONCLUSION

Colchicine can be effective in reducing systemic symptoms of COVID-19 by inhibiting inflammatory biomarkers.

## Data Availability

Not applicable.

## ABBREVIATIONS

ARDS: Acute respiratory distress syndrome
COVID-19: Coronavirus Disease 2019
ICU: Intensive care unit
IL: Interleukin
